# Methylation associated inactivation of RASSF1A and its synergistic effect with activated K-Ras in nasopharyngeal carcinoma

**DOI:** 10.1186/1756-9966-28-160

**Published:** 2009-12-30

**Authors:** Tao Wang, Hongli Liu, Yeshan Chen, Wei Liu, Jing Yu, Gang Wu

**Affiliations:** 1 Cancer Center of Wuhan Union Hospital, Tongji Medical College, Huazhong University of Science and Technology, Wuhan, 430022, China; 2 The Department of Oncology, Tongji Hospital, Tongji Medical College, Huazhong University of Science and Technology, Wuhan, China

## Abstract

**Background:**

Epigenetic silencing of tumor suppressor genes associated with promoter methylation is considered to be a hallmark of oncogenesis. RASSF1A is a candidate tumor suppressor gene which was found to be inactivated in many human cancers. Although we have had a prelimilary cognition about the function of RASSF1A, the exact mechanisms about how RASSF1A functions in human cancers were largely unknown. Moreover, the effect of mutated K-Ras gene on the function of RASSF1A is lacking. The aim of this study was to investigate the expression profile and methylation status of RASSF1A gene, and to explore its concrete mechanisms as a tumor suppressor gene in Nasopharyngeal Carcinoma.

**Methods:**

We examined the expression profile and methylation status of RASSF1A in two NPC cell lines, 38 primary nasopharyngeal carcinoma and 14 normal nasopharyngeal epithelia using RT-PCR and methylated specific PCR(MSP) respectively. 5-aza-dC was then added to confirm the correlation between hypermethylation status and inactivation of RASSF1A. The NPC cell line CNE-2 was transfected with exogenous pcDNA3.1(+)/RASSF1A plasmid in the presence or absence of mutated K-Ras by liposome-mediated gene transfer method. Flow cytometry was used to examine the effect of RASSF1A on cell cycle modulation and apoptosis. Meanwhile, trypan blue dye exclusion assays was used to detect the effect of RASSF1A transfection alone and the co-transfection of RASSF1A and K-Ras on cell proliferation.

**Results:**

Promoter methylation of RASSF1A could be detected in 71.05% (27/38) of NPC samples, but not in normal nasopharyngeal epithelia. RASSF1A expression in NPC primary tumors was lower than that in normal nasopharyngeal epithelial (*p *< 0.01). Expression of RASSF1A was down-regulated in two NPC cell lines. Loss of RASSF1A expression was greatly restored by the methyltransferase inhibitor 5-aza-dC in CNE-2. Ectopic expression of RASSF1A in CNE-2 could increase the percentage of G0/G1 phase cells (*p *< 0.01), inhibit cell proliferation and induce apoptosis (*p *< 0.001). Moreover, activated K-Ras could enhance the growth inhibition effect induced by RASSF1A in CNE-2 cells (*p *< 0.01).

**Conclusion:**

Expression of RASSF1A is down-regulated in NPC due to the hypermethylation of promoter. Exogenous expression of RASSF1A is able to induce growth inhibition effect and apoptosis in tumor cell lines, and this effect could be enhanced by activated K-Ras.

## Background

Nasopharyngeal carcinoma (NPC) is a serious and common cancer in Southern China. The tumorigenesis of NPC is a multistage process involving cellular genetic predisposition, epigenetic alterations, including the influence of environment factors, diet and Epstein-Barr virus (EBV) infection[1,2]. However, the molecular basis leading to the development and spread of NPC remain largely unknown. Recent years, several studies [3,4] showed that silence of tumor suppressor genes by epigenetic modification is a major mechanism for inactivation of cancer-related genes in the pathogenesis of human cancers. Cheng et al. reported that epigenetic events, including DNA methylation and chromatin structure changes, are among the earliest molecular alterations during malignant transformation of human mammary epithelial cells[5]. Methylation of the CpG islands of DNA promoter is the most important and common epigenetic mechanism leading to gene silence[6]. Consequently, identification of genes targeted by hypermethylation may provide insight into NPC tumorigenesis.

A numer of tumor suppressor genes have been implicated to harbor promoter methylation at CpG islands in NPC, such as RASSF1A (Ras association domain family 1 isoform A), p16, BLU [7,8] and recently LARS2 (leucyl-tRNA synthetase 2, mitochondrial) was found to involve in this process[9]. RASSF1A inactivation is essential for tumor development. Moreover, there is growing evidence demonstrating that a high frequency of methylation associated inactivation of the tumor suppressor gene RASSF1A was detected in a wide range of human cancers [10-13].

Since methylation of the RASSF1A promoter is described as an early and frequent event in tumorigenesis, it could serve as a useful diagnostic signal in cancer screens. Previous studies suggested that RASSF1A may implicate in various cellular mechanisms including cell cycle arrest, apoptosis, inhibition of cell proliferation *in vitro *[14-17] as well as repression of tumor formation in nude mice [18], however, little is known about the underlying mechanisms of RASSF1A. The most interesting structure feature of RASSF1A proteins is the presence of a Ras association (RA) domain, which determines the role of RASSF1A protein functions as a Ras-effector, and endows RASSF1A the ability to interact with Ras family protein[18]. The Ras proteins are intimately involved in the regulation of a wide variety of biological processes by interacting with different downstream effectors. Although it is widely accepted that the Ras functions as an oncoprotein that contribute to cell proliferation through the RAS-MAP-kinase pathway and antiapoptotic effect, more and more studies found that it also induces growth arrest of cells, such as apoptosis and senescence by interact with specific effectors [19]. RASSF1A, act as a newly discovered downstream negative effector of Ras protein, may interact with Ras protein in a GTP-dependent manner and induce a potent, Ras-mediated apoptosis [20].

In this study, we characterized the hypermethylation status of promoter of RASSF1A in NPC tumor biopsies and normal nasopharyngeal epithelia. Growth inhibition effect including cell cycle arrest, apoptosis and senescence was also observed in CNE-2 cells that were transfected with exogenous RASSF1A gene. Furthermore, we have initiated to figure out whether this tumor suppression effect of RASSF1A could be enhanced in the presence of activated Ras.

## Materials and methods

### NPC cell lines and tissue samples

Two NPC cell lines, CNE1 and CNE2 were maintained in RPMI 1640 supplemented with 10% fetal bovine serum at 37°C. A total of 38 primary tumor biopsies cases were obtained from newly diagnosed and untreated NPC patients with consent and 14 samples of normal nasopharyngeal epithelial tissues were obtained from the suspected patients as normal controls at the department of otolaryngology at the Union Hospital of Tongji Medical College (Wuhan, China). All of the specimens were subjected to histological diagnosis by pathologists according to the WHO classification. Relative data involving age, gender, clinical stage, lymph node metastasis and distance metastasis were collected after the patients visiting. High-molecular weight DNA was extracted from the samples using DNA extract kit (Tiangen) according to the manufacture's instructions.

### RT-PCR

Total RNAs from cell lines, normal nasopharyngeal epithelia and tumor biopsies was isolated with TriZOL regent (Huashun biotechnology). One micrograms of total RNA was used for the first strand synthesis of cDNA by ReverTra Ace™ Kit (TOYOBO, Janpan). The PCR was carried out in a total volume of 25 μl PCR reaction containing 10 pmol of each primer, 2.5 μl of deoxy-ribonucleoside triphosphate, 1 × PCR buffer, 1 unit of Taq polymerase (Fermantas) and 2 μl of template cDNA. The primer sequences used for amplification of RASSF1A were 5'-CTTTTACCTGCCCAAGGA TGC-3' and 5'-CACCTCCCCAGAGTCATTTTC-3'. The primers for GAPDH (5'-CATGACAACTTTGGTATCGTG-3' and 5'-GTGTCGCTGTTGAAGTCGTCAG A-3') were used as internal control, and the annealing temperature was 55°C for RASSF1A and 58°C for GAPDH. After 25 cycles, 8 μl of PCR products were loaded onto a 1.5% agarose gels, stained with GoldView, and visualized under UV illumination.

### Sodium bisulfite modification

High-molecular weight genomic DNA from primary tumor biopsies and normal nasopharyngeal epithelial tissues were subjected to bisulfite modification by using the CpGenome™ DNA Modification Kit (Chemicon International, USA) according to the manufacture's instruction; Treatment of genomic DNA with sodium bisulfite converts unmethylated cytosines, but not methylated cytosines to uracil, which is then converted to thymidine during the subsequent methylated specific PCR steps [21].

### Methylated specific PCR

The methylation status of RASSF1A promoter region was detected by methylated-specific PCR assay, PCR primers that distinguishing unmethylated (U) and methylated (M) DNA sequences were described by Burbee et al.[22]. The primers used to detect the methylated form were 5'-GGGTTTTGCGAGAGCGCG-3'(forward) and 5'-GCTAACAAACGCGAACCG-3'(reverse), and the primers to detect the unmethylated form were 5'-GGTTTTGTGAGAGTGTGTTTAG-3' (forward) and 5'-CACTAACAAACACAAACCAAAC-3' (reverse). Each primer set generated a 169-bp product. Genomic DNAs, modified by bisulfite treatment, were used as a template for methylated specific PCR (MSP). Each MSP reaction incorporated 2 μl of sodium bisulfite-modified DNA, 10 pmol of each primer, 2.5 μl of deoxy-ribonucleoside triphosphate, 1 × PCR buffer, MgCl_2 _and 1 unit Taq polymerase (Fermantas) in a final PCR reaction volume of 25 μl. The annealing temperature was 64°C for methylation-specific and 59°C for unmethylation-specific primers. DNA modified by methylase Sss I was used as a positive control and water was included as negative control. The PCR products were separated on 2% agarose gels stained with GoldView fluorochrome (Saibaisheng) and visualized under UV illumination.

### 5-Aza-2'-deoxycytidine treatment

To determine whether RASSF1A expression could be restored by the demethylating agents, the NPC cell line CNE-2, which showed to have lower expression of RASSF1A than CNE-1 in our studies, was subjected to 5-aza-2'-deoxycytidine treatment. 2 × 10^5 ^CNE-2 cells were plated in a six-well plate and incubated for 4 d with 0, 1, 3, 5, 7, 10 μmol/L 5-aza-2'-deoxycytidine (Sigma). The medium and drug were replaced every 24 h. The re-expression and alteration of methylation status of RASSF1A in NPC cell line CNE-2 after 5-aza-dC treatment was examined by RT-PCR and MSP as described previously in our study.

### Plasmids and transfection

Growth inhibition assays were performed by transiently transfecting CNE-2 cells with 3 μg of pcDNA3.1(+)/RASSF1A construct (a generous gift from Prof. Reinhard Dammann, Department of Biology, Beckman Research Institute, City of Hope Medical Center, Duarte, California, USA.) or pcDNA3.1(+) empty vector using Lipofectamine 2000 (Invitrogen, USA). pCGN-HA-RasG12V (a generous gift from Prof. Geoffrey J. Clark, Department of Cell and Cancer Biology, National Cancer Institute, Rockville, Maryland, USA.), which contains the cDNAs encoding activated K-Ras gene, was used to perform co-transfection with pcDNA3.1(+)/RASSF1A in CNE-2 cells. Transfection was performed using Lipofectamine 2000 (Invitrogen, USA) according to the manufacturer's instruction. The expression of exogenous RASSF1A and K-RasG12V was confirmed by RT-PCR analysis and western-bloting.

### Western-blot analysis

Cells were grown and harvested at 70-80% confluency, cellular protein were extracted with lysis buffer which contains PMSF, a protease inhibitors (BOSTER), Lysates were incubated on ice for 30 min, and insoluble cell debris was removed by centrifugation for 10 min at 12,000 rpm at 4°C. Protein samples were separated by 10-15% SDS-PAGE and were electroblotted to PVDF membranes (Roche) and stained with enhanced chemiluminescence solution. For detection of bound primary antibody, the membranes were then incubated with the mouse monoclonal anti-RASSF1A (eBioscience). β-actin protein level were used as a control for equal protein loading.

### Cell death assay

CNE-2 cell death assays were performed by transfection cells with 4 μg each of empty vector or pcDNA3.1 (+) RASSF1A in the presence or absence of 40 ng of K-Ras12V. Briefly, 1.5 × 10^5 ^CNE-2 cells were seeded in 6-well plates one day before transfection, 48 h post-transfection, trypan blue was added *in situ *at a final concentration of 0.04%. Dead cells were quantitated by counting the number of blue cells in three random 40 × field using phase/contrast microscopy.

### Cell cycle analysis

Cell cycle analysis was performed in CNE-2 cells after the treatment of 5-aza-dC for 4 d and transfected with 3 μg of pcDNA3.1 (+)/RASSF1A or empty vector using Lipofectamine 2000. Four days after agent treatment and 48 h after transfection, cells were harvested and fixed in ice-cold 70% ethanol at 4°C overnight. Then cells were washed twice with ice-cold PBS and pelleted by centrifugation and the ethanol was decanted. Cells were resuspended at a concentration of 1 × 10^6 ^cells/ml in staining solution (65 μg/ml propidium iodide, 50 μg/ml RNase A). After incubation at 37°C in dark for 30 min, cells were subjected to flow cytometry (FACSort) analysis. Cellular DNA content was assessed and cell cycle model was acquired.

### Apoptosis assays

CNE-2 cells were transfected with 4 μg of RASSF1A in the presence or absence of 40 ng of K-RasG12V or empty vector using Lipofectamine 2000. Annexin-V binding was used to measure apoptosis. 1 × 10^5 ^cells were seeded in 6-well dishes. 48 h post-transfection, cells were harvested using trypsin, washed with ice-cold PBS, resuspended in 500 μl annexin-V binding buffer and incubated at room temperature with 5 μl of each of Annexin-V and Propidium Iodide (Annexin V-FITC apoptosis detection kit; NanJing KeyGen Biotech. Co. LTD) for 15 min in dark. Then, a FACSort flow cytometer was used to measure Annexin-V-PI binding.

### Statistical analysis

Statistical analysis was performed by software package SPSS 13.0. All experiments were repeated independently, at least three times. Values are given as means ± SD. The possible correlation between methylation status and clinicopathological features were analysis using Pearson Chi-Square test. RASSF1A expression level in NPC primary tumors compared to normal nasopharyngeal epithelia and RASSF1A-methylated tumors compared to unmethylated tumors were analysis by using Mann-Whitney's U test. *P *< 0.05 was considered to be statistically significant.

## Results

### Expression of RASSF1A in NPC cell lines and nasopharyngeal biopsy specimen

**Figure 1:**
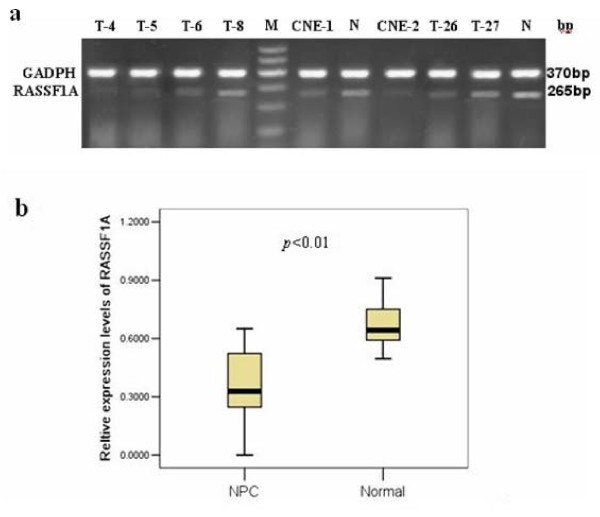
**(a) Expression level of RASSF1A in NPC cell lines, normal nasopharyngeal epithelial and primary tumor biopsies by RT-PCR, T, primary nasopharyngeal tumor tissues; N, normal nasopharyngeal epithelial; M; marker I**. GAPDH was amplified as an internal control. **(b) **Summary of overall expression of RASSF1A in 38 primary NPC tumors and 14 normal nasopharyngeal epithelial biopsies. RASSF1A expression was significantly down-regulated in NPC primary tumors compared with normal nasopharyngeal epithelial (*p *< 0.01, Mann-Whitney's U test).

The two NPC cell lines had a low expression level of RASSF1A and all of the normal nasopharyngeal epithelial biopsies expressed an easily detectable level of RASSF1A. The overall expression of RASSF1A in 38 primary NPC tumors was down-regulated compared to that of 14 normal nasopharyngeal epithelial biopsies (*p *< 0.01), and with completely silenced of RASSF1A expression in 2 cases of primary NPC tumors (Figure 1).

### Hypermethylation of RASSF1A in NPC cell lines, primary tumorsand normal nasopharyngeal epithelia

**Figure 2:**
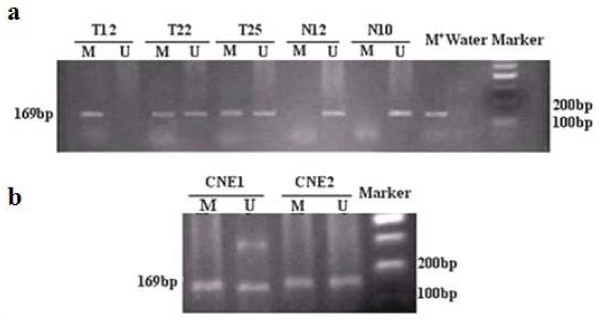
**(a) Methylation-specific PCR analysis of RASSF1A promoter region in NPC primary tumors and normal nasopharyngeal tissues**. Three NPCs (T12, T22, T25) and two normal nasopharyngeal (N12, N10) were showed as examples. DNA modified by methylase SssI severed as a positive methylation control and water was included as blank control. M: methylated alleles; U: unmethylated alleles. **(b) **Methylation status of RASSF1A in NPC cell lines CNE-1 and CNE-2, DNAs from these two cell lines could be amplified with both methylated (M) and unmethylated (U) DNA-specific primers.

Promoter hypermethylation of RASSF1A could be detected in 71.05% (27/38) of the primary NPC tumors but not in the normal NP epithelia (Figure 2a). MSP analysis of RASSF1A promoter in NPC cell lines, CNE-1, CNE-2 is shown in Figure 2b. DNAs from the two cell lines could be amplified with both methylated and unmethylated DNA-specific primers. This result revealed that these two cell lines were partial methylation.

### Inactivation of RASSF1A correlates with its hypermethylation

**Figure 3:**
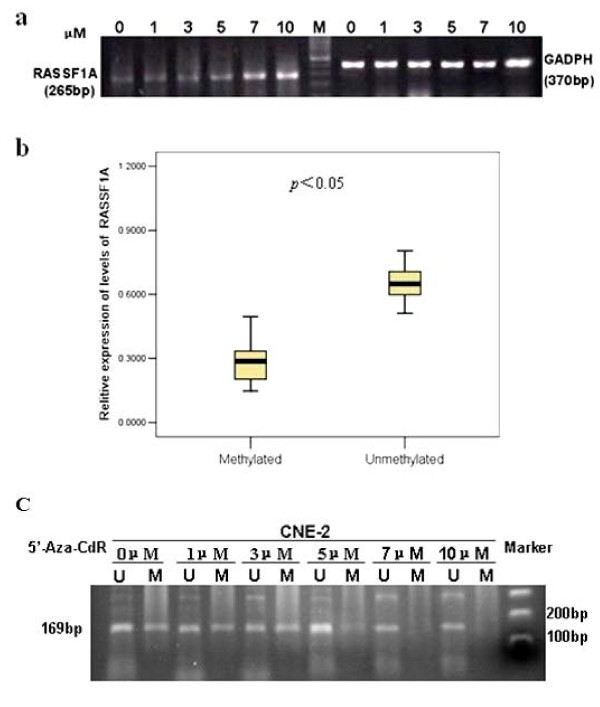
**(a) Re-expression of RASSF1A by treatment with 5-aza-2'-deoxycytidine in CNE-2 cell lines at different concentration (0, 1, 3, 5, 7, 10 μmol/L), and GAPDH was amplified as an internal control**. **(b) **Summary of RASSF1A expression in RASSF1A-methylated and--unmethylated NPC primary tumors. Inactivation of RASSF1A expression was significantly correlated with promoter hypermethylation of RASSF1A (*p *< 0.05, Mann-Whitney's U test). **(c) **The methylation status of RASSF1A after the treatment of 0, 1, 3, 5, 7, 10 μmol/L of 5-aza-2'-deoxycytidine in CNE-2 cells.

Based on the RT-PCR result and MSP analysis, methylation of RASSF1A could be detected in 2 NPC cell lines in which RASSF1A expression were down-regulated. The normal nasopharyngeal epithelial biopsies, which have a normal expression level of RASSF1A, presented only unmethylated alleles. Additionally, a decreased level of RASSF1A expression could be detect in RASSF1A-methylated 27 primary NPC cases compared to unmethylated NPC cases (*p *< 0.05, Figure 3b).

To further demonstrate that promoter hypermethylation contributes to the lack of expression of RASSF1A in the NPC cell lines, we assessed the effect of 5-aza-2'-deoxycytidine, a drug that inhibits DNA methylation. CNE-2 had lower expression of RASSF1A than CNE-1 had in our studies. So CNE-2 was chosen and treated with 0, 1, 3, 5, 7, or 10 μmol/L of 5-aza-dC for 4 d. We observed that the re-expression level of RASSF1A was gradually up-regulated alone with the increase of drug concentration (Figure 3a), but little change could be observed in the expression of the internal control gene GAPDH. Then the methylation status of RASSF1A in each concentration groups showed that the groups of 0, 1, 3, 5 μmol/L showed amplification for both methylated and unmethylated sequences, but in the groups of 7 and 10 μmol/L of 5-aza-dC treatment, only unmethylated alleles could be detected (Figure 3c).

### Clinicopathological significance of RASSF1A promoter hypermethylation

A significant correlation was observed between the frequency of promoter hypermethylation of RASSF1A and the differentiation degree of the tumor (χ^2 ^= 4.932, *p *< 0.05), but no correlation was observed between promoter methylation of RASSF1A and the patients' age, gender, clinical stage, lymph node metastasis or distance metastasis (*p *> 0.05) (Table 1).

**Table 1:** Correlation between RASSF1A promoter methylation and clinicopathological index in NPC

	No. of patient	Promoter methylation status		Frequency of methylationincidence	
				
		Methylated	Unmethylated		
Gender					NS
Male	22	17	5	77.27%	
Female	16	10	6	62.50%	
Age					NS
≤50	17	14	3	82.35%	
>50	21	13	8	61.90%	
Histological subtype					*p *= 0.047
poorly differentiated	27	22	5	81.48%	
Well-differentiated	11	5	6	45.45%	
Stage					NS
I and II	13	9	4	69.23%	
III and IV	25	18	7	72.00%	
Lymph node					NS
Positive	29	22	7	75.86%	
Negative	9	5	4	55.56%	
Distance metastasis					NS
Positive	5	4	1	80.00%	
Negative	33	23	10	69.70%	

### Exogenous expression of RASSF1A and K-Ras synergistically inhibits cell growth

**Figure 4:**
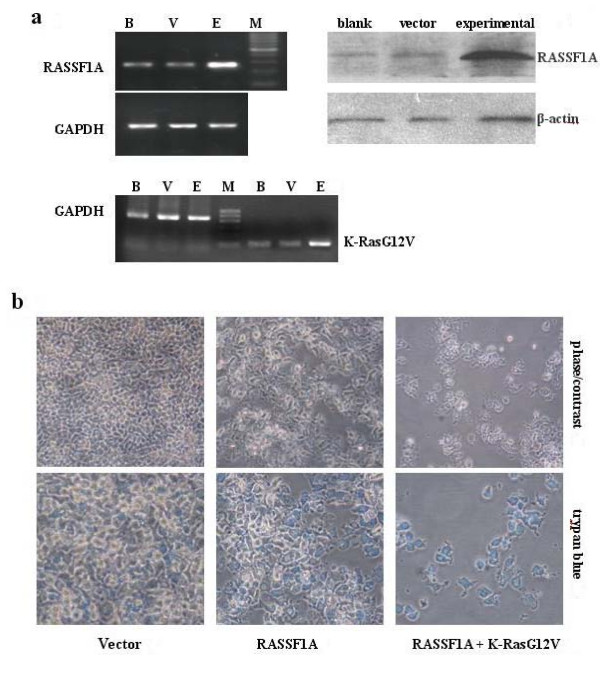
**RASSF1A-mediated growth inhibition and cell death is enhanced by K-RasG12V**. CNE-2 cells were transiently transfected with RASSF1A ± activated K-Ras. Trypan blue was added *in situ *after 48 h, and the dye uptake was quantitated. **(a) **Transfect efficiency of RASSF1A and K-RasG12V is confirmed by RT-PCR and western-blot. B: blank group, V: empty vector group, E: experimental group;**(b) **Cell death assays; up-panel: CNE-2 cells were transfected with RASSF1A ± K-RasG12V, phase contrast microscopic digital images were taken at 48 h post-transfection, RASSF1A promoted a modest growth inhibition that was enhanced by the presence of activated K-RasG12V; lower-panel: Trypan blue *in situ *staining, the dye uptake was enhanced when RASSF1A was co-expressed with activated K-Ras.

**Figure 5:**
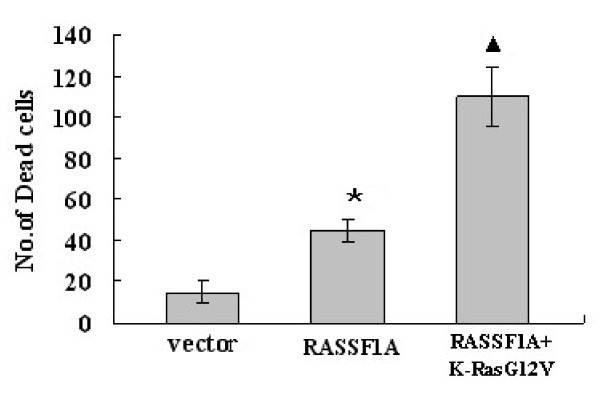
**Quantification analysis of the result of cell death assay is the average of three experiments**. *: vs Vector group, *p *< 0.001; (Black triangle): vs RASSF1A group, *p *< 0.01.

To determine the growth inhibition effect of RASSF1A, CNE-2 cells were transfected with RASSF1A ± activated K-Ras, the transfect efficiency was measured by RT-PCR and western-blot analysis respectively (Figure 4a). After examined for 48 h, modest growth inhibition was detected with RASSF1A alone, but this effect was dramatically enhanced by the presence of activated K-Ras (Figure 4b). We observed that RASSF1A on its own promoted modest cell death as the amount of blue dead cells were less. But in the presence of activated K-Ras12V, the dead blue cells were enhanced greatly (*p *< 0.01, Figure 5). It seems that co-transfection of these two genes together could induced synergistic cell death effect.

### RASSF1A mediate cell cycle arrest and Ras-dependent apoptosis

**Figure 6:**
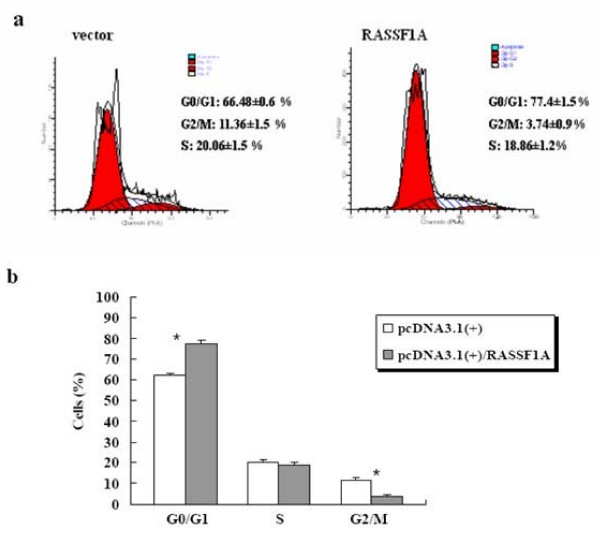
**Ectopic expression of RASSF1A induces cell cycle arrest**. **(a) **Cell cycle arrest effect of RASSF1A, the CNE-2 cells were transiently transfected with either empty vectors or RASSF1A-expression vectors, after 48 h, the CNE2-RASSF1A cells showed a 11% increase in G0/G1 phrase cells than CNE2-empty vector cells. **(b) **The statistical analysis of the cell cycle distribution. *: vs Vector group, p < 0.01.

48 h post-transfection, analysis of propidium iodide incorporation of the RASSF1A-expression CNE-2 cells showed an 11% increase in G0/G1 phase cell population than that of empty vector expression CNE-2 cells (*p *< 0.01) (Figure 6).

**Figure 7:**
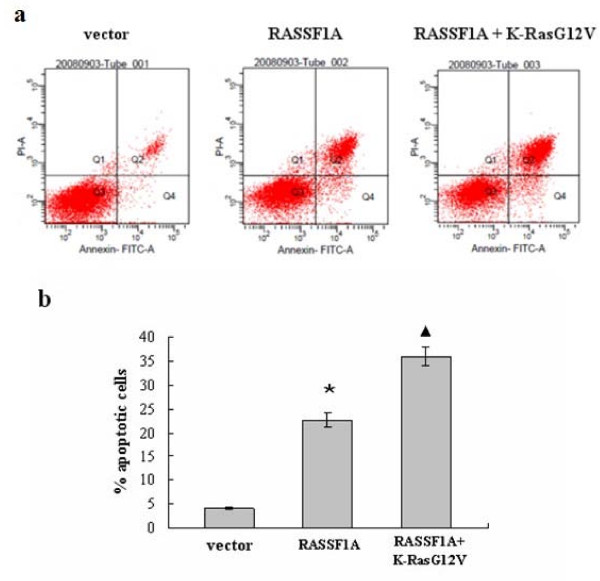
**RASSF1A promotes apoptosis that is enhanced by K-RasG12V**. **(a) **CNE-2 cells were transiently transfected with empty vector and RASSF1A-expression vectors in the presence or absence of activated K-Ras, 48 h post transfection, empty vector cells showed 4.1% of apoptosis rate, RASSF1A expression cells was 22.7%, and RASSF1A + activated K-Ras expression CNE-2 cells showed 36.0% of apoptosis rate. **(b) **The statistical analysis of the apoptotic cells in each group. *: vs Vector group, p < 0.001; (Black triangle): vs RASSF1A group, p < 0.001.

What's more, compared to the empty vector, RASSF1A on its own could promote apoptosis, but activated Ras(G12V) dramatically stimulated this apoptosis effect (*p *< 0.001)(Figure 7). Thus, based on this result, we believed that ectopic expression of RASSF1A could inhibit tumorigenicity through induction of cell cycle arrest in G0/G1 phase, and mediated apoptosis in a Ras-dependent manner.

## Discussion

Recent studies concerned with epigenetic research are mostly focus on the RASSF1 family, which has three major transcripts, A, B and C. The transcript A and C were expressed in all normal tissues, but RASSF1A expression was impaired in a number of lung tumor cell lines and in several other cancer cell lines [10,11]. Loss of expression was correlated with methylation of the CpG-island promoter sequence of RASSF1A. Reintroduction of RASSF1A in SCLC lines reduces colony formation, suppressed anchorage-independent growth and inhibited tumor formation in nude mice[18]. Moreover, it was reported that Rassf1a-knockout mice are apt to suffer from various cancers[23]. These characteristics lead to a proposal that the RASSF1A isoform is the major tumor suppressor gene inactivated in many kinds of tumors by promoter methylation, which is the major mechanism for inactivation of RASSF1A since an observation of point mutation in RASSF1A gene was found to be a rare event in a majority of human cancers[24]. Chow et al. and Steinmann et al. demonstrated that RASSF1A is a critical tumor suppressor gene harboring with high frequency of promoter methylation, which is located on 3p21.3 in NPC[13,25].

In our study, we detected that RASSF1A mRNA expression was down-regulated in NPC cell lines and primary tumors. Methylation specific PCR and RT-PCR analysis also revealed a correlation between RASSF1A expression level and methylation status in NPC cell lines, primary tumors and normal epithelial. 5-aza-2'-deoxycytidine treatment further confirmed that promoter hypermethylation contributes to the lack of expression of RASSF1A in the NPC cell lines. Base on these findings, hypermethylated DNA could be served as a potential molecular tumor marker that distinguishes cancers from normal tissues.

Our MSP analysis showed that RASSF1A methylation was frequent in NPC, as the RASSF1A promoter region was subjected to methylation in 71.05% of the primary tumors, the two NPC cell lines that we examined were also both partial methylation. In addition, our findings of a lack of RASSF1A methylation in the normal nasopharyngeal epithelia support the fact that epigenetic silencing of RASSF1A is a tumor specific process. Moreover, hypermethylation of RASSF1A can be detected in both early-stage and advanced NPC tumors, suggesting that RASSF1A gene promoter methylation might play an important role in the early development of nasopharyngeal carcinogenesis.

Although the fact that a high frequency of promoter hypermethylation of RASSF1A that function as a tumor suppressor is widely accepted by many researchers, and the growth inhibition effect of RASSF1A in CNE-2 cells was observed by trypan blue dye exclusion assays in our present studies. However, the regulation and mechanism of action of RASSF1A remain a topic of intense investigation [26]. It appears that like many other critical tumor suppressors, RASSF1A is multifunctional, thus, inactivation of RASSF1A may impact many different facets of tumor biology. *In vitro *expression of RASSF1A in H1299 lung carcinoma cells inhibited cell cycle progression by negatively regulating the accumulation of cyclin D1 through a posttranscriptional mechanism [27]. It was reported that RASSF1A overexpression in gastric carcinoma cell lines led to a cell cycle arrest at G1 phase, and activator protein-1(AP-1) is necessary for this process[28]. A recent research indicated that SKP-2, an oncogenic subunit of an ubiquitin ligase complex, which founctions as a critical regulator of S phase progression, could promote degradation of RASSF1A at the G1/S checkpoint and then lead to the cell cycle proceeding in hepatocellular carcinoma[29]. In our study, we further confirmed the ability of RASSF1A to induce cell cycle arrest in NPC cell line CNE-2. Furthermore, RASSF1A was found to be capable of inducing apoptosis in our result although it was not observed by some other study[27]. Previous studies indicated that there are several different apoptotic pathways that RASSF1A is said to be involved in. It was observed by Vos et al. that RASSF1A can activate Bax via MOAP-1(a Bax binding protein) and activated K-Ras, thus, RASSF1A and MOAP-1 synergize to induce Bax activation and cell death[17]. Also, RASSF1A was found to invovled in death receptor-dependent apoptosis through MOAP-1. Upon tumor necrosis factor α (TNF-α) stimulation, MOAP-1 associates with the TNF receptor 1, subsequently, RASSF1A was recruited to this complex and then participates in the death receptor-dependent apoptosis[30].

The Ras-signaling pathway also plays an important role in tumorigenesis. Although Ras oncoproteins were initially characterized as suppressor of apoptosis, it is now clear that they also have the ability to promote apoptosis and inhibit proliferation, that serve as a protective mechanism[19]. The Ras family proteins are a group of membrane-bound small GTPase which comprise 21 members such as H-Ras, K-Ras and N-Ras. As a negative effector of Ras, RASSF1A may shift the balance of Ras signaling pathway toward a cell growth inhibition including senescence, apoptosis and cell cycle arrest. Several studies have confirmed the ablilty of RASSFs family to interact with different Ras family proteins. RASSF1A is a pro-apoptosis protein that has a potential Ras association (RA) domain, which makes the RASSF1A has the theoretical potential to bind to Ras directly *in vitro*, and form a complex with activated K-Ras when overexpressed in cells[31,32]. This suggests that it may function as an effector for Ras. However, some authors have failed to see direct binding between Ras and RASSF1A, they suggest that the interaction is indirect or RASSF1A alone binds only weakly to Ras protein due to heterodimerization of RASSF1A with NORE1[33]. But RASSF2, another member of RASSFs family, is thought to possess the ability to bind directly to K-Ras in a GTP-dependent manner via its RA domain[34]. In our studies, we have hypothesized that RASSF1A may serve as an effector that mediate Ras-associated growth inhibition effect, including Ras-dependent apoptosis. Consequently, to examine the potential modulation of RASSF1A activity by Ras, we decided to measure the consequence of activated K-Ras12V expression on RASSF1A-induced growth arrest of human nasopharyngeal carcinoma cell lines. The expression of mutated K-Ras which is an activated form of this gene is rare in nasopharyngeal carcinoma but is common in some other tumor types, with as high as 90% in pancreatic carcinomas, 30% in NSCLC [35]. As we could observed, RASSF1A has an endogenous ability to promote apoptosis in CNE-2 cells, however, this activity is indeed dramatically stimulated by activated K-Ras in nasopharyngeal carcinoma cell lines CNE-2, which is contrast to the observations by Shivakumar *et al *in mammary adenocarcinoma cells[27]. Although we were unable to explore the concrete association mechanism between RASSF1A and activated Ras, synergistic effect of the co-expression of the two genes could be confirmed by cell death assays and apoptosis analysis. These data leading to the possibility that Ras may positively regulate the activity of endogenous RASSF1A. In addition, a mutual exclusion between RASSF1A inactivation by methylation and K-Ras mutation was observed in a number of human cancers such as pancreatic cancer and endometrial carcinoma[36,37], supporting the association of RASSF1A with the Ras signaling pathways.

Nasopharyngeal carcinoma is a radiosensitive cancer. The early-diagnosed patients who receive the treatment of radiotherapy with or without chemotherapy would accquire a high curative rate. A reliable molecular marker need to be identified to diagnose and predict the progression and prognosis of NPC. It was reported by Chang et al. that a high detection rate of tumor surpressor genes such as RASSF1A could be evaluated in peripheral blood, mouth and throat rinsing fluid and nasopharyngeal swabs of NPC patients, indicating the potential role of epigenetic events in non-invasive screening of NPC[38]. Moreover, inactivation of RASSF1A was found to be correlated with lymph node metastasis[39] and tumor stage in NPC[8], however, it was not observed in our group. These investigations support the possibility of methylation of tumor suppressor genes could be a sensitive marker in diagnosing and predicting nasopharyngeal carcinogenesis. Although hypermethylation of the promoter sequence is the major mechanism that leads to inactivation of tumor suppressor genes, fortunately, this modified process could be reversed as there is no alterations on the gene sequences, employment of the demethylated agent 5-aza-2'-deoxycytidine could induce the recovery of the function of these tumor suppressor gene [18] and it indeed happened in NPC. This suggests that alteration of the epigenetic changes of the gene would be a new way of tumor therapy.

## Conclusion

In summary, the expression of RASSF1A was markedly reduced or completely lost in primary nasopharyngeal carcinoma compared with normal nasopharyngeal epithelia, and was correlated to hypermethylation of the promoter of the RASSF1A gene. The tumor suppressor function of this gene involved in cell cycle arrest, inhibiting cell proliferation and inducing apoptosis. Furthermore, our study confirmed that these growth-inhibitory properties could be enhanced by activated K-Ras, although the physiological interaction between Ras and RASSF1A has yet to be elucidated. Further studies are needed to be focused on understanding the molecular mechanism of RASSF1A activity. In a word, RASSF1A represents an important potential diagnostic and therapeutic target and the loss or inactivation of RASSF1A may be a critical component of the evolution of Ras-dependent tumors.

## Competing interests

The authors declare that they have no competing interests.

## Authors' contributions

WT and WG supervised the design of the experiments and analysed and interpreted of data. LHL conceived the study and helped to draft the manuscript. CYS was involved in the cell transfection, Western-blotting, Cell death and Apoptosis assays, Cell cycle analysis, drafting of the manuscript and design of the study. LW carried out the Bisulfate modification and MSP studies, drug intervention study and performed the statistic analysis. YJ contributed to the collection of biopsy samples and clinical data and carried out the RT-PCR. All authors have read and approved the final manuscript.
